# Meis2 controls skeletal formation in the hyoid region

**DOI:** 10.3389/fcell.2022.951063

**Published:** 2022-09-28

**Authors:** Jaroslav Fabik, Viktorie Psutkova, Ondrej Machon

**Affiliations:** ^1^ Department of Developmental Biology, Institute of Experimental Medicine of the Czech Academy of Sciences, Prague, Czechia; ^2^ Department of Cell Biology, Faculty of Science, Charles University, Prague, Czechia

**Keywords:** Meis2, cartilage, hyoid bone, Hand2, PBX1, mesenchymal condensation

## Abstract

A vertebrate skull is composed of many skeletal elements which display enormous diversity of shapes. Cranial bone formation embodies a multitude of processes, i.e., epithelial-mesenchymal induction, mesenchymal condensation, and endochondral or intramembranous ossification. Molecular pathways determining complex architecture and growth of the cranial skeleton during embryogenesis are poorly understood. Here, we present a model of the hyoid apparatus development in Wnt1-Cre2-induced *Meis2* conditional knock-out (cKO) mice. *Meis2* cKO embryos develop an aberrant hyoid apparatus—a complete skeletal chain from the base of the neurocranium to lesser horns of the hyoid, resembling extreme human pathologies of the hyoid-larynx region. We examined key stages of hyoid skeletogenesis to obtain a complex image of the hyoid apparatus formation. Lack of *Meis2* resulted in ectopic loci of mesenchymal condensations, ectopic cartilage and bone formation, disinhibition of skeletogenesis, and elevated proliferation of cartilage precursors. We presume that all these mechanisms contribute to formation of the aberrant skeletal chain in the hyoid region. Moreover, *Meis2* cKO embryos exhibit severely reduced expression of PBX1 and HAND2 in the hyoid region. Altogether, MEIS2 in conjunction with PBX1 and HAND2 affects mesenchymal condensation, specification and proliferation of cartilage precursors to ensure development of the anatomically correct hyoid apparatus.

## Introduction

A vertebrate skull, especially the facial skeleton, exhibits enormous diversity of skeletal shapes, and the basis behind this variety lies within skeletal development. Skeletogenesis is a stepwise set of processes with each step depending on the step before. Early craniofacial skeletogenesis involves neural crest (NC) migration, epithelial-mesenchymal induction, and condensation. On the other hand, late-stage skeletogenesis commonly includes differentiation, deposition of extracellular matrix, and terminal differentiation (Hall and Miyake, 1995, 2000; Hall, 2015a, 2015b, 20; Giffin et al., 2019). Despite the fact that early skeletogenic events are absolutely essential for the position, size, and number of skeletal elements, they have been considerably overlooked by researchers (Hall and Miyake, 1995, 2000). While differentiation of mesenchymal precursors into chondroblasts or osteoblasts is fairly described on the molecular level, little is still known about early stages of skeletogenesis. To this date, skeletogenesis has been best described in long bones of limbs. Nevertheless, cranial bone formation, which includes some of the most complex shapes in the vertebrate skeleton, is less understood. Both mandibular and hyoid arches give rise to sets of complex structures, practically forming the facial skeleton (Frisdal and Trainor, 2014; Poopalasundaram et al., 2019; Fabik et al., 2021). However, less is known about skeletogenesis in the hyoid arch than in the mandibular arch. Similar to the mandibular apparatus, the hyoid bone is a very ancient structure, that is homologous to the ceratohyal cartilage in fish species (Kitazawa et al., 2015). In mammals, the hyoid apparatus facilitates swallowing, breathing, and sound production, and interestingly, there is an evidence that mammalian hyoid bones appeared in the mammalian lineage even before the separation of the middle ear and mandible (Zhou et al., 2019).

In humans and mice, the hyoid apparatus encompasses the styloid processes (SP), stylohyoid ligaments, lesser horns of the hyoid (LH), body, and greater horns of the hyoid bone. Together with laryngeal cartilages and its ligaments, it forms the hyoid-larynx complex (de Bakker et al., 2018, 2019). Cartilage elements of the hyoid apparatus arise in the hyoid arch and in the third pharyngeal arch (PA3), whereas according to new research, the body of the hyoid forms as a single *de novo* mesenchymal condensation in the midline (Rodríguez-Vázquez et al., 2011; de Bakker et al., 2018). The hyoid arch gives rise to the cranial styloid process and caudal lesser horns of the hyoid (Rodríguez-Vázquez et al., 2006). During early hyoid development in humans, the SP and LH are connected via band of mesenchymal tissue that is thought to differentiate into stylohyoid muscles and ligaments (Rodríguez-Vázquez et al., 2015). The PA3 gives rise to the greater horns of the hyoid, and possibly also to superior horns of the thyroid cartilage (Rodríguez-Vázquez et al., 2011).

The hyoid apparatus goes through identical stages of skeletal development as the rest of pharyngeal skeletal elements. Initially, all mesenchyme is uniform and seemingly homogenous. Mesenchymal condensations are initiated by epithelial-mesenchymal interactions. For example, the formation of the avian basihyal bone (homologous with the mammalian hyoid body) depends on the patterning activity of the ventromedial zones of the foregut endoderm (Ruhin et al., 2003). It is widely believed that during the condensation phase, the hyoid-specific morphology is tuned via the patterning activity of *Hox* genes in the neural crest of the hyoid and the PA3. Early condensations, including the condensation for a hyoid primordium, are aggregations of densely-packed cells, that are characteristic of a close contact between cells and sparse or absent extracellular matrix (Hall, 2015a). The close contact is ensured by expression of cell adhesion molecules like *N-cadherin* (Cdh2), which also acts to establish cell communication via gap junctions (Rundus et al., 1998; DeLise and Tuan, 2002). Additionally, *tenascin-C* and *syndecan3* are expressed at the boundaries of condensation and subsequently set off the definitive boundary from the surrounding non-skeletogenic mesenchyme, which later becomes a perichondrium, or a periosteum, respectively (Koyama et al., 1995, 1996) (Hall, 2015a).

The central dogma of the condensation growth is that skeletogenic condensations must reach a critical size, otherwise the normal process of chondrogenesis does not occur (Hall and Miyake, 2000). This is achieved by cellular mechanisms that affect mesenchymal cells both inside and outside the condensation—these include cell proliferation, cell death, migration of cells towards the center or absence of cell movement away from the center (Hall and Miyake, 1992; Hall and Miyake, 1995; Hall and Miyake, 2000; Franz-Odendaal, 2011). The last two are recognized as the predominant mechanisms of skeletogenic condensation growth for the majority of species (Franz-Odendaal, 2011). Abnormally large condensations will result in cartilage formation in places where no cartilage is normally present. Mutations in *Hoxa2*, *Dlx2*, and *Prrx1* produce ectopic cartilages in mice (Gendron-Maguire et al., 1993; Rijli et al., 1993; Martin et al., 1995; Qiu et al., 1995, 2), indicating abnormal condensations during craniofacial development. Concurrently with condensation growth, patterning of condensations in shape, number, arrangement, and location takes place and establishes the future skeletal morphology (Karsenty, 2003; Eames et al., 2012). Little is known about patterning of condensations in the hyoid arch and what is known mostly comes from studies primarily concerning the mandibular arch. The distal part of the styloid process is controlled by *Edn1-Dlx5/Dlx6* regulatory cascade; while *Dlx1/Dlx2* controls the proximal part, similarly to the mandibular arch. Therefore, the boundary between proximal and distal hyoid arch lies in the styloid process. Worth of note, mouse mutants with mandibular anomalies often exhibit defects in the hyoid-larynx complex, suggesting a common regulatory circuit during development of the mandibular and hyoid arch (Fabik et al., 2021). The idea of this common regulatory circuit is further supported by transplantation studies, where mouse dental epithelium can organize dental papilla formation in the mouse hyoid arch (Mina and Kollar, 1987).

Transition from condensation to overt cell differentiation into osteoblasts and chondroblasts requires down-regulation of genes controlling cell adhesion and proliferation (Hall, 2015b). Naturally, progression to the differentiation phase requires up-regulation of genes associated with differentiation. RUNX2 is widely regarded as the osteogenic transcription factor, just as SOX9 is widely regarded as the key chondrogenic transcription factor. Consequently, lack of *Sox9* in the neural crest results in loss of all cartilage elements derived from the cranial neural crest (Mori-Akiyama et al., 2003, 9), while lack of *Runx2* leads to total agenesis of bone (Komori et al., 1997). The balance between the two determines, whether osteo- or chondrogenesis will be initiated from competent mesenchymal cells (Hall, 2015b). A second important osteogenic transcription factor SP7 acts downstream of RUNX2. While RUNX2 regulates transformation of progenitor cells to preosteoblasts, SP7 is required for transformation of preosteoblasts to osteoblasts and has a function in switching cells from chondrogenesis to osteogenesis (Hall, 2015b). Both SOX9 and RUNX2 regulate osteo- and chondrogenesis by up-regulating the expression of extracellular matrix genes—SOX9 upregulates *Col2a1* and *Col11a2* (Bell et al., 1997; Lefebvre et al., 1997), while RUNX2 upregulates *osteocalcin* and *osteopontin* (Komori, 2006). Epithelial signals seem to be involved in differentiation of cells into osteoblasts and chondroblasts as well. SHH appears to be necessary for cartilage differentiation, and thereby proper formation of the mandible, by regulating *Col1a*, *Sox9*, and *Col2a* expression (Billmyre and Klingensmith, 2015).

Novel mechanisms of cartilage growth and shaping during osteochondrogenic differentiation have been proposed by Kaucka et al., 2017. Pharyngeal cartilages are formed as bars or rods (i.e., Meckel’s, the styloid process, the lesser and greater horns of the hyoid), and diameter control of pharyngeal cartilages is achieved through formation of clonal columns (Kaucka et al., 2017). Additionally, the mechanism controlling the diameter of pharyngeal cartilages also involves the regulation of cell number within each chondrogenic clone. Therefore, the shape of a pharyngeal cartilage could be regulated by differentiation speed via impinging on the clone size. Meanwhile, chondrogenic condensations at the very tip of a pharyngeal cartilage enable elongation. Complementary to microgeometries and clonal domains, tuning of macro-geometry could be achieved through a stage-specific placement of proliferative hot zones where new clonal domains intercalate into the main cartilage structure. Dynamic distribution of fast and slow proliferative cartilage progenitors results in creation of localized growth zones. These provide for the general cartilage expansion but may also bend the cartilage by creating local tensions that require mechanical relaxation and influence further development of the overall shape (Schötz et al., 2013). Another way to fine-tune macro-geometry of cartilaginous structures is continuous adding of pre-shaped chondrogenic mesenchymal condensations from the pool of competent progenitors. Such mechanisms could operate in the developing hyoid, as new chondrogenic condensations are responsible for introducing geometrically complicated patterns in the facial skeleton (Kaucka et al., 2017).

de Bakker et al., 2019 have shown that the hyoid-larynx complex is a subject of great diversity in humans. Variants of the hyoid-larynx complex have an incidence of 4–30% (Shul’ga et al., 2006; Petrović et al., 2008; De Paz et al., 2012; Naimo et al., 2015). However, only less than 10% of these manifest clinically, among which the Eagle’s syndrome and the aberrant hyoid apparatus are diagnosed most commonly. (Chiang et al., 2004; Petrović et al., 2008; De Paz et al., 2012). Eagle’s syndrome, also known as the stylohyoid syndrome, describes a collection of clinical symptoms related to anomalous elongation and angulation of the styloid process, which disturbs surrounding anatomical structures (Shul’ga et al., 2006; Petrović et al., 2008; De Paz et al., 2012). Unlike the Eagle’s syndrome, the aberrant hyoid apparatus affects the entire stylohyoid chain, not just the styloid process, forming a complete bony chain from the base of the skull to the hyoid bone (Lykaki and Papadopoulos, 1988; Vougiouklakis, 2006). Many authors suggested that this chain is derived from transient embryonic Reichert’s cartilage.

In this paper, we carefully examined formation of the hyoid apparatus in the *Meis2* conditional knock-out (cKO) mice. *Meis2* cKO has proven to be an excellent mouse model for the study of skeletal formation in the pharyngeal arches, as lack of *Meis2* affects derivatives of the mandibular arch, i.e., the mandible and palate (Machon et al., 2015; Fabik et al., 2020; Wang et al., 2020). In addition to mandibular arch anomalies, *Meis2* cKOs also exhibit previously unreported hyoid arch anomalies. Therefore, we focused on key stages of skeletogenesis in the hyoid region to obtain a complex image of the hyoid apparatus formation. Together, we examined mesenchymal condensation, mesenchymal progenitor proliferation, differentiation, and even the positional architecture of dividing cells, all of which influence the shape of the hyoid apparatus. Finally, we unraveled a gene regulatory circuit involving MEIS-HAND-PBX that controls skeletal formation in the hyoid region.

## Material and methods

### Mouse breeding

Generation of the floxed allele of *Meis2* gene (*Meis2* ^f/f^) with loxP sites around exons 2–6 was described in Machon et al. (2015). Generation of the floxed allele of *Meis1* gene (*Meis1* ^f/f^) was described in Fabik et al. (2020). *Meis2* ^f/f^ and *Meis1* ^f/f^ strains were crossed to R26R-*EYFP* ^f/f^ (The Jackson Laboratory strain #006148) before crossing to the Wnt1-Cre2 strain to monitor the tissue specificity of Cre recombination in the craniofacial area. Wnt1–Cre2 (The Jackson Laboratory strain #022137) was used for specific deletion of the *Meis2* ^f/f^ gene in neural crest cells. Our standard crossing scheme for generating *Meis2* cKO embryos was: the male Wnt1-Cre2; *Meis2* ^f/+^ and the female *Meis2* ^f/f^; R26R-*EYFP* ^f/f^ (similarly *Meis1* cKO). During embryo harvesting, we always checked Cre recombination by monitoring EYFP fluorescence. In very rare cases (1–2 embryos in 10 litters), we observed Cre activity in the whole body indicating germline recombination. Such animals were removed from the analysis and crossing schemes. *Meis1* ^f/f^, *Meis2* ^f/f^, R26R-*EYFP* ^f/f^ were maintained in C57BL/6J background. Original stock Wnt1-Cre2 was created in 129S4/SvJaeJ background but it was further maintained in C57BL/6J in our animal facility.

Mice were mated overnight and the day of the vaginal plug observed was regarded as the embryonic day 0.5 (E0.5). Pregnant mice were anaesthetized with isoflurane gas, euthanized by cervical dislocation, and embryos were harvested. All procedures involving experimental animals were approved by the Institutional Committee for Animal Care and Use (permission #PP-10/2019). This work did not include human subjects.

### Immunohistochemistry

Embryos were harvested at desired stages and then fixed in 4% paraformaldehyde (PFA) in phosphate buffered saline (PBS) overnight at 4°C. Embryos were washed in PBS and either:

i) dehydrated through graded series of ethanol, cleared in xylene and embedded in paraffin;
or
ii) incubated in 30% sucrose overnight, and embedded in OCT.

Paraffin (6 µm) or frozen sections (10 µm) were permeabilized in 0.1% Tween-20 in PBS (PBT). Antigen retrieval was carried out in 0.1 M citrate buffer pH 6.0 under pressure boiling for 12 min. Sections were blocked in 5% bovine serum albumin (BSA) for 2 h and incubated overnight with a primary antibody diluted in 1% BSA in PBT. After washing in PBT, sections were stained with a biotinylated secondary antibody, Vectastain ABC Elite Kit and 3,3′-diaminobenzidine (DAB). In case of fluorescent Alexa secondary antibodies, nuclei were counterstained with DAPI (4,6-diamino-2-phenylindol, 0.1 μg/ml, Roche). Primary antibodies: Hand2 (1/1000) (Abcam ab2000040), Meis1 1/750) (Atlas Antibodies HPA056000), Meis2 (1/2000) (Abnova H004212), Pbx1 (1/500) (Cell Signalling 4342S) Runx2 (1/1000) (Abcam ab192256), Sox9 (1/2000) (Milipore AB5535), Sp7 (1/1000) (Abcam ab22552).

### Quantification of cell proliferation and dividing cluster orientation

Pregnant mice were injected with 5-ethynyl-2′-deoxyuridine (EdU) (total 1.25 mg in 0.125 ml PBS per mouse) intraperitoneally 24 h prior to the embryo harvest. Embryos were fixed in 4% PFA in PBS overnight at 4°C, washed in PBS and incubated in 30% sucrose overnight. Thick frozen sections (150 µm) were stained whole-mount with a primary antibody diluted in 1% BSA and 0.5% Triton-X-100 in PBS at 4°C for 3 days, washed, and incubated with an Alexa secondary antibody for 2 days. Sections were washed in PBT and stained overnight using EdU BaseClick kit according to manufacturer’s instructions. After washing in PBT, sections were mounted on a microscopy glass and scanned by spinning disc confocal microscope Andor Dragonfly 503. Quantification of double positive cells was performed in control littermates and *Wnt1-Cre2; Meis2* ^*f/*f^ embryos. Three embryos of each genotype and stage were used for cell quantifications in orthogonal projections of 150 μm-thick sections. SOX9/EdU-double positive and RUNX2/EdU-double positive cells were counted in hyoid primordia. Percentages were calculated from the numbers of double positive cells related to the total number of SOX9-or RUNX2-positive cells in hyoid primordia. Values are presented as averages with standard deviations. To assess EdU-positive 2-cell cluster orientation, we quantified doublets oriented in three arbitrary angles towards the longitudinal axis. 90° represent perpendicular while 0° mean parallel orientation towards the longitudinal hyoid axis. For simplicity, 4-cell clusters were regarded as two 2-cell clusters. Percentages were calculated from multiple z-stack images of 150-μm thick sections from biological triplicates.

### *In situ* hybridization

cDNAs were cloned into pGEM-T-easy vector (Promega) using primers: Prrx1 F- CTC​CTA​TTA​AGT​GGA​GAT​CTG​C, Prrx1 R- AAC​AGA​AGA​AGG​CTT​GTT​CC, Dlx5 F- TAG​ACC​AGA​GCA​GCT​CCA​CA and Dlx5 R- CTG​TAG​TCC​CAA​AAC​TGA​GC.

*Hoxa2* probe plasmid was kindly provided by Professor Abigail Saffron Tucker. Antisense mRNA was transcribed with T7 or SP6 polymerase. Whole-mount *in situ* hybridization was performed using standard protocols (Machon et al., 2002; Wei et al., 2011).

### Skeletal preparations

Embryos were incubated in water for 1 h at 4°C. Afterwards they were scalded in boiling water for 5 min and they were skinned. They were dehydrated in 95% ethanol for 72 h, and the ethanol was exchanged every 12 h. Alcian blue (Sigma-Aldrich) was used to stain cartilage for 12 h, afterwards embryos were rinsed twice in fresh ethanol and incubated in ethanol overnight. Embryos were cleared in 1% KOH for 2 h, and then stained with Alizarin Red S (Sigma-Aldrich) for 5 h. Excess stain was then rinsed off with fresh 1% KOH for 1 h and further clearing in 1% KOH was carried out overnight. Embryos were transferred through a graded series of glycerol (25%) and KOH (1%) for 8 h, and glycerol (50%) and KOH (1%) for 48 h. Pictures were obtained using binocular microscope Olympus SYX9 and camera Olympus DP72.

## Results

### *Meis2* is strongly expressed in the hyoid region and *Meis2* cKOs exhibit hyoid arch anomalies

**FIGURE 1:**
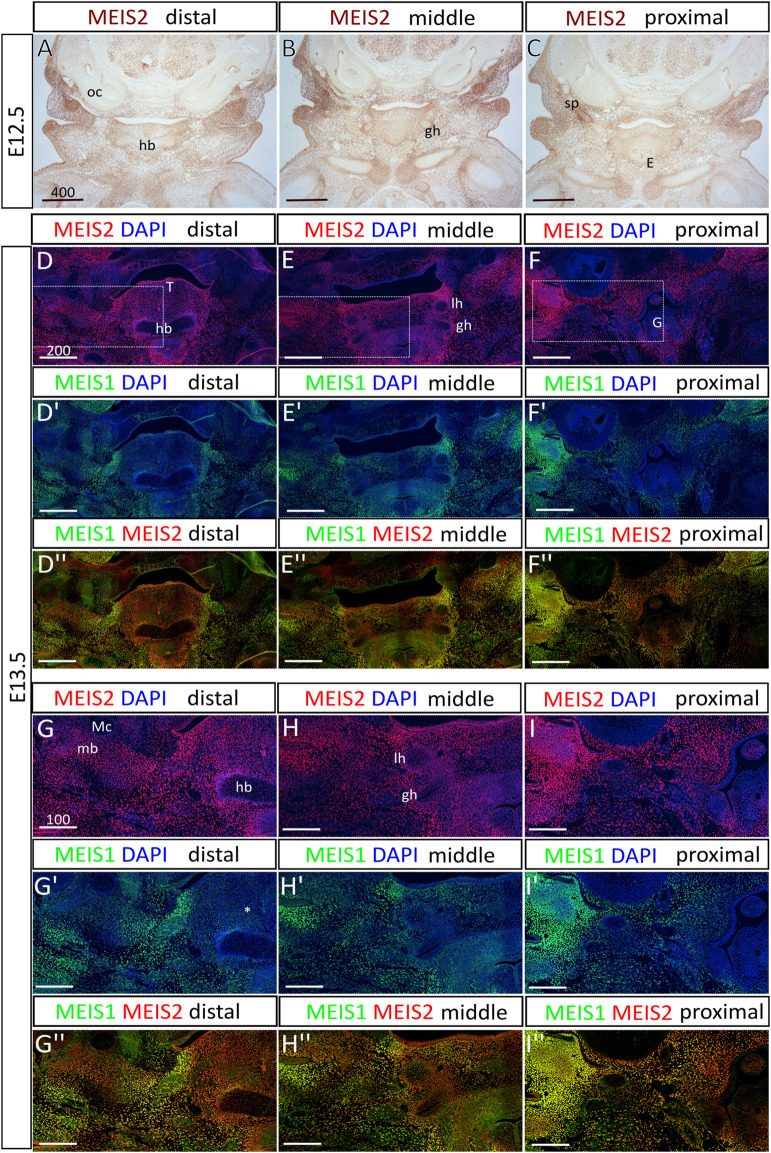
The expression of *Meis2* in the embryonic hyoid region. **(A–C)** Frontal paraffin sections of E12.5 hyoid regions. MEIS2 protein is abundant in non-skeletogenic mesenchyme of the hyoid region and in chondrocyte precursors in and around the hyoid primordium. MEIS2 signal is weaker in the hyoid body, while strong in the lesser horns, the greater horns and the styloid process. **(D–F")** MEIS1 and MEIS2 double immunofluorescence showing significantly weaker MEIS1 signal in the hyoid cartilage, surrounding mesenchyme and the tongue. **(G–I")** Higher magnification images showing MEIS1 and MEIS2 double immunofluorescence of boxed areas in **(D–F)**. Abbreviations: hb, hyoid body; gh, greater horns; lh, lesser horns; mb, mandible; Mc, Meckel’s cartilage; oc, otic capsule; sp, styloid process; E, epiglottis; G, glottis; T, tongue. Scale bars in μm.

Previously, we have reported that MEIS2 transcription factor is abundantly expressed in cranial NC cells, regulates molecular patterning of the mandibular arch, and is necessary for osteochondrogenic differentiation in the facial skeleton. To analyze skeletal development in the hyoid region, we used a mouse conditional knockout model with Wnt1-Cre2-driven ablation of *Meis2* ^f/f^ in the neural crest (*Meis2* cKO). First, we mapped the spatiotemporal pattern of *Meis2* expression in the hyoid region at embryonic days (E) 12.5 and 13.5 (Figure 1). We observed strong MEIS2 signal in mesenchyme of the hyoid region and in developing chondrocytes of the prospective hyoid apparatus. In E12.5 embryos (Figures 1A–C), we found that MEIS2 signal appeared weaker in the hyoid body, although it remained strong in the greater horns and in the styloid process. Similarly, in E13.5 embryos (Figures 1D–F), we observed a weaker MEIS2 staining in the hyoid body, while markedly stronger signal was detected in the surrounding mesenchyme, in the greater horns, in the lesser horns and the styloid process. In contrast, MEIS1, the paralogue of MEIS2, stained much weaker in the hyoid cartilage with almost no expression in the central hyoid mesenchyme (asterisk in Figure 1G′), and tongue. Lateral regions of the mandibular and hyoid arch around the oral cavity showed overlapping signal of MEIS1 and MEIS2 (Figure 1D–I″). It is also worth of note that *Meis2* is strongly expressed in the tongue epithelium and developing mandible around Meckel’s cartilage while *Meis1* is not (Figure 1D, D″). We conclude that *Meis2* is expressed in developing chondrocytes of the hyoid apparatus, the hyoid perichondrium and in surrounding non-skeletogenic mesenchyme of the hyoid region. In contrast, *Meis1* expression appeared remarkably weaker in these areas.

**FIGURE 2:**
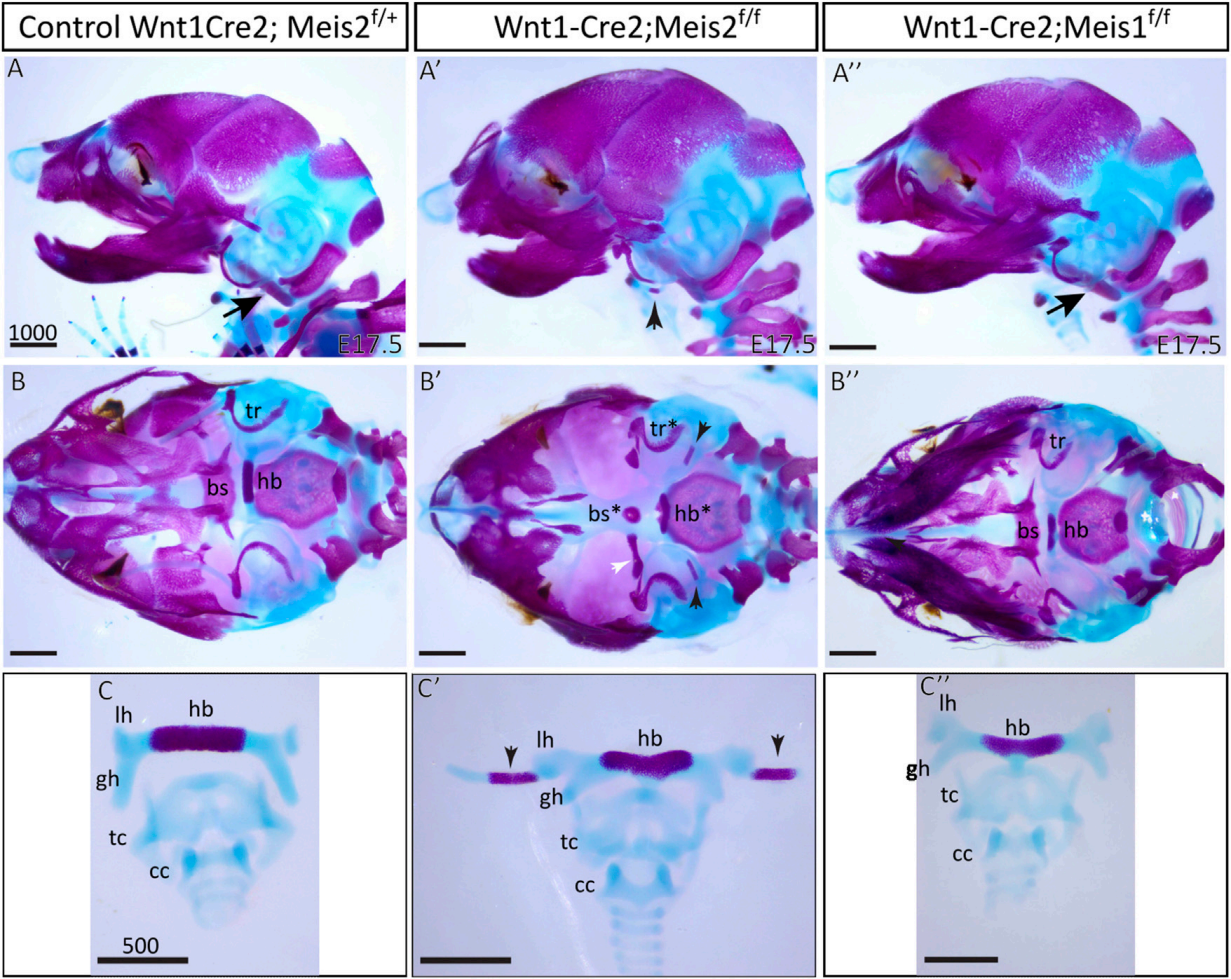
Craniofacial and hyoid phenotypes in control, Wnt1-Cre2; *Meis2* ^f/f^ and Wnt1-Cre2; *Meis1* ^f/f^ embryos. **(A–A")** Lateral view at skeletal preparations of heads of E17.5 control, *Meis2* cKO and *Meis1* cKO embryos. **(B–B")** Inferior view at skeletal preparations of heads of E17.5 control, *Meis2* cKO and *Meis1* cKO embryos. **(C–C")** Frontal view at skeletal preparations of hyoid-larynx complexes of E17.5 control, *Meis2* cKO and *Meis1* cKO embryos. In *Meis2* cKO, observe an aberrant skeletal chain that stretches from the styloid process to the lesser horns of the hyoid (black arrowheads in **A′,B′,C′**). Arrows in A and A″ point at normal styloid process. Note an extra bony element close to the gonial bone, the malleus, and the tympanic ring (white arrowhead in **B′**). Abbreviations: hb, hyoid body; gh, greater horns; lh, lesser horns; sp, styloid process (arrows in **A** and **A″"**); tc, thyroid cartilage; cc, cricoid cartilage; bs, basisphenoid bone; tr, tympanic ring. Scale bars in μm.

Skeletal preparations of the hyoid apparatuses in E17.5 *Meis2* cKO embryos revealed many severe anomalies in formation of the cranium—including anomalies in the basisphenoid bone (bs*), tympanic rings (tr*), and in the hyoid apparatus (hb*), in all tested mutant embryos (8 out of total 8 embryos, 8/8). Specifically, an aberrant skeletal chain linking the styloid process and the lesser horns of the hyoid (Figure 2A′,B′,C′; black arrowheads) was observed in mutants. This aberrant skeletal chain was partially mineralized (Figure 2B′–C′; arrowheads). Additionally, we observed an extra skeletal element of undetermined origin close to the gonial bone, the malleus and the tympanic ring (Figure 2B′, white arrowhead). In contrast, *Meis1* cKO generated by the same Wnt1-Cre2 driver displayed normal development of the hyoid, basisphenoid bone and tympanic rings (Figure 2A"–C"). Unaffected development of these skeletal structures in *Meis1* cKO may be explained by weaker or hardly detectable *Meis1* expression at earlier stages (see Figure 1). For purpose of this study, we chose only the hyoid apparatus formation in *Meis2* cKO for further analysis.

### Ectopic mesenchymal condensations precede the aberrant hyoid apparatus formation in the hyoid region of *Meis2* cKOs

**FIGURE 3:**
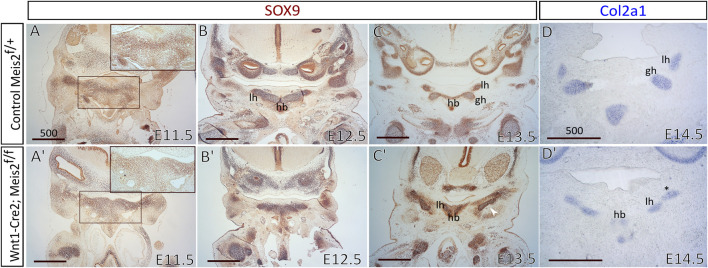
The expression of *Sox9* in the embryonic hyoid region. Frontal paraffin sections of hyoid regions in control and Wnt1-Cre2; *Meis2* ^f/f^ embryos. **(A, A′)** See a clear SOX9^+^ outline of the hyoid primordium in E11.5 control **(A**, inset**)**, whereas SOX9^+^ cells in E11.5 Wnt1-Cre2; *Meis2* ^f/f^ did not form any distinctively recognizable shape **(A′**, inset**)**. **(B,B′)** The cartilage primordium of the hyoid bone formed in its normal shape in E12.5 control, while the hyoid primordium in E12.5 Wnt1-Cre2; *Meis2* ^f/f^ was still represented by persisting SOX9^+^ chondrogenic condensations. **(C,C′)** Persisting SOX9^+^ chondrogenic condensations were also observed in E13.5 Wnt1-Cre2; *Meis2* ^f/f^. Note an extra skeletal element close to the gonial bone, the malleus, and the tympanic ring (white arrowhead in **C′**). **(D,D′)** *Col2a1 in situ* hybridization showing the expression in the hyoid primordium and the aberrant skeletal chain (asterisk) in E14.5 control and Meis2 cKO. Abbreviations: hb, hyoid body; gh, greater horns; lh, lesser horns. Scale bars in μm.

To elucidate the molecular origin of this defect, we focused on cartilage development in the nascent hyoid apparatus through several phases. Chondrocytes were stained with SOX9 antibody on paraffin sections. Already in E11.5 embryo (Figure 3A), we noticed a clear SOX9^+^ outline of the hyoid primordium in the control (Figure 3A, the inset), whereas SOX9^+^ cells in *Meis2* cKO did not organize themselves into any distinctively recognizable form (Figure 3A’ and the inset). In the control E12.5 embryo, the cartilage primordium of the hyoid bone appeared already formed in a normal shape (Figure 3B). In the E12.5 *Meis2* cKO (3/3), we observed persisting SOX9^+^ chondrogenic condensations (Figure 3B′) creating a highly SOX9^+^ outline of the hyoid primordium that was not noted in the E11.5 *Meis2* cKO (Figure 3A′). In the E13.5 *Meis2* cKO, the cartilage primordium of the hyoid bone already formed, however, in comparison with controls, the primordium did not acquire a normal shape and we found persisting SOX9^+^ chondrogenic condensations surrounding the cartilage template (Figure 3C, C′). We presume that SOX9^+^ chondrogenic condensations in the hyoid region of Meis2 cKOs represent ectopic mesenchymal loci that are prerequisites for the aberrant hyoid apparatus, including the chain. Prior to E11.5, SOX9 is a marker of migrating cranial neural crest cells that will differentiate into NC-derived cranial chondrogenic precursors. In the control hyoid arch, first signs of chondrogenic condensations were noticed at E11.5. To investigate chondrogenic differentiation, we monitored collagen, type II, alpha 1 expression by *in situ* hybridization on sections using *Col2a1* riboprobe. As seen in Figure 3D,D′, *Col2a1* was normally expressed in the malformed cartilage hyoid primordium in the *Meis2* cKO, suggesting that early phases of chondrogenic differentiation were not affected in mutants.

**FIGURE 4:**
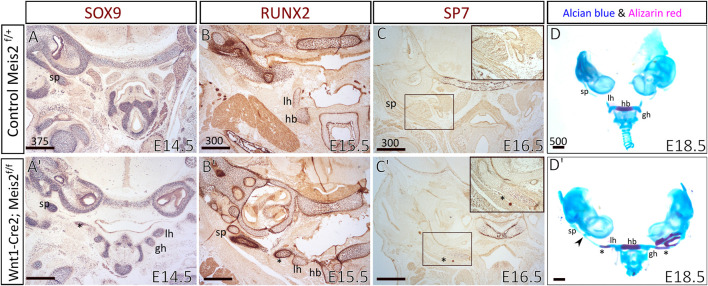
The aberrant skeletal chain between the lesser horns and the styloid process in Wnt1-Cre2; Meis2 ^f/f^. Frontal paraffin sections **(A–C′)** of the hyoid apparatuses in controls and Wnt1-Cre2; *Meis2* ^f/f^. **(A,A′)** In E14.5 Wnt1-Cre2; *Meis2* ^f/f^, SOX9-staining revealed the aberrant skeletal chain (asterisk) that was close to the lesser horns and was continuous with the styloid process. **(B,B’)** In E15.5 Wnt1-Cre2; *Meis2* ^f/f^, RUNX2-staining revealed an intensive staining of the aberrant skeletal chain (asterisk). **(C,C′)** In E16.5 Wnt1-Cre2; *Meis2* ^f/f^, SP7-staining revealed commitment of the aberrant skeletal chain (asterisk) to the osteoblast lineage. **(D,D′)** Frontal view at skeletal preparations of the hyoid-larynx complexes with temporal bones. E18.5 Wnt1-Cre2; *Meis2* ^f/f^ specimens have developed the aberrant hyoid apparatus. A small caudal part of the aberrant skeletal chain is mineralized (asterisk in **D′**) and a cartilaginous nodule randomly formed along the chain (black arrowhead in **D′**). Abbreviations: hb, hyoid body; gh, greater horns; lh, lesser horns; sp, styloid process; tc, thyroid cartilage; cc, cricoid cartilage. Scale bars in μm.

To examine the process of formation and mineralization of the aberrant skeletal chain, we analyzed the expression of osteochondrogenic markers in the distal region of the hyoid apparatus, using SOX9, RUNX2, and SP7 antibodies. We compared E14.5–16.5 hyoid regions between controls and *Meis2* cKOs. In E14.5 *Meis2* cKO (6/6), we observed the aberrant skeletal chain (Figure 4A′, asterisk) close to the lesser horns of the hyoid and the chain was continuous with the styloid process (Figure 4A′). In the E15.5 *Meis2* cKO, we found strong RUNX2 signal in the aberrant cartilage (Figure 4B′, asterisk) and the lesser horns (Figure 4B′). In the E16.5 *Meis2* cKO, the aberrant cartilage (Figure 4C′, asterisk) stained positive for SP7, indicating commitment of the aberrant skeletal chain to the osteoblast lineage (Figure 4C′) (2/2). We could not find any SP7^+^ cells in the aberrant skeletal chain in the E15.5 *Meis2* cKO embryo (data not shown). Therefore, we presume that, in addition to the effect on mesenchymal condensation, lack of *Meis2* also affects differentiation of chondrocytes into osteoblasts in the caudal part of the aberrant skeletal chain. Based on the domain of SP7 expression and Alizarin staining, the part of the aberrant skeletal chain mineralizes in *Meis2* cKO embryos between E15.5-16.5. Upon skeletal preparations, we dissected hyoid apparatuses along with temporal bones in E18.5 fetuses (Figure 4D-D′). We again observed the mineralized aberrant skeletal chain in *Meis2* cKOs (5/5) (Figure 4D′, asterisk), and we also noticed ectopic cartilaginous nodules randomly adjacent to the chain in one whole-mount specimen (black arrowhead in 4D′). In 7/8 analyzed mutant specimen at E13.5 or later, the hyoid apparatus acquires abnormal shape, growing a downward projection in the midline (Figures 2C′ and Figure 3C′). This further supports our histological data that ectopic mesenchymal loci appear between lesser horns and the styloid process, and give rise to the aberrant ectopic cartilage, later forming the aberrant hyoid apparatus. To sum up, ectopic mesenchymal condensations give rise to the aberrant skeletal chain and a part of the skeletal chain mineralizes.

### Elevated proliferation and uneven distribution of chondrogenic precursors in the hyoid primordium of *Meis2* cKOs

**FIGURE 5:**
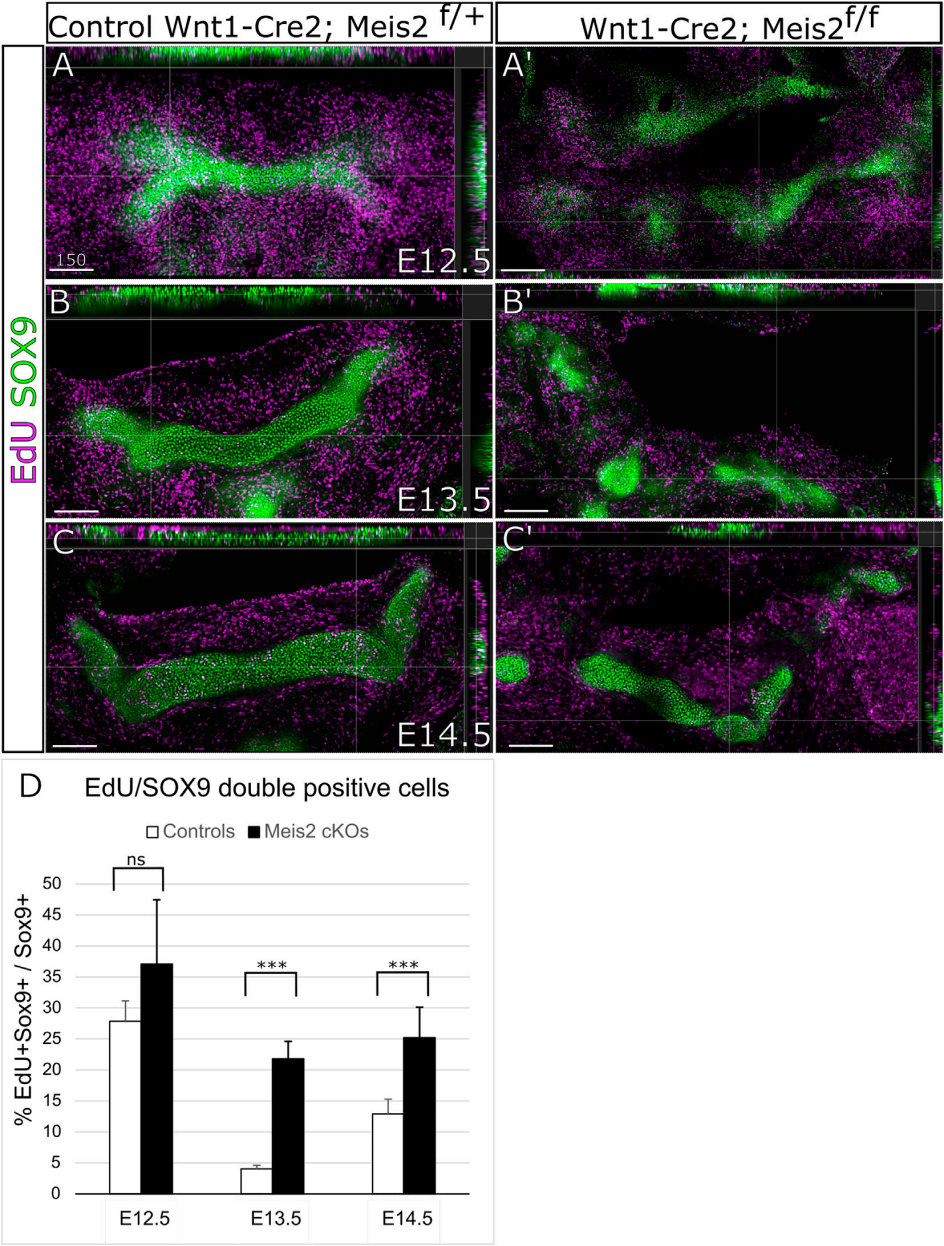
Elevated proliferation of SOX9 cells in the cartilage primordium of the hyoid bone in Wnt1-Cre2; *Meis2* ^f/f^. **(A–C′)** Frontal frozen sections with orthogonal projections of hyoid regions in controls and Wnt1-Cre2; *Meis2* ^f/f.^. **(D)** Quantifications of the percentage of double positive EdU/SOX9 cells in controls and Wnt1-Cre2; *Meis2* ^f/f^ (E12.5 control 27.88 ± 3.27); (E12.5 Wnt1-Cre2; *Meis2* ^f/f^ 37.19 10.32); (E13.5 control 3.84 ± 0.70); (E12.5 Wnt1-Cre2; Meis2^f/f^ 22.20 ± 2.78); (E14.5 control 12.90 ± 2.41); (E14.5 Wnt1-Cre2; *Meis2* ^f/f^ 25.24 ± 4.90) Statistical analysis was performed from biological triplicates for each genotype using unpaired two-tailed Student’s t-test. Scale bars in μm.

Next, we wanted to elucidate the cellular mechanism causing abnormal growth and abnormal formation of the hyoid apparatus. Therefore, we assessed cell proliferation in the cartilage and perichondrium of the hyoid primordium. We analyzed cell proliferation in the hyoid cartilage primordium by EdU incorporation and SOX9 labelling. In parallel, cell proliferation in the perichondrium was examined by EdU incorporation and RUNX2 staining. As shown in Figure 5A,A′, around 30% chondrocytes in the hyoid primordium proliferate (EdU^+^/SOX9^+^ double positives). The difference in EdU^+^/Sox9^+^ double positive cells in E12.5 embryos was not statistically significant between controls and *Meis2* cKOs. Nonetheless, the chondrogenic condensation in E12.5 *Meis2* cKO did not take the hyoid shape as distinct as the one in the control. In E13.5 and E14.5 embryos, we observed significantly more proliferating chondrocytes in *Meis2* cKOs (Figure 5B-C′). Quantifications from orthogonal projections of three mutants and controls are summarized in Figure 5D. These results suggest that elevated proliferation of SOX9^+^ cartilage progenitors may account for cartilage and chondrogenic condensation growth in the mutant hyoid primordium.

**FIGURE 6:**
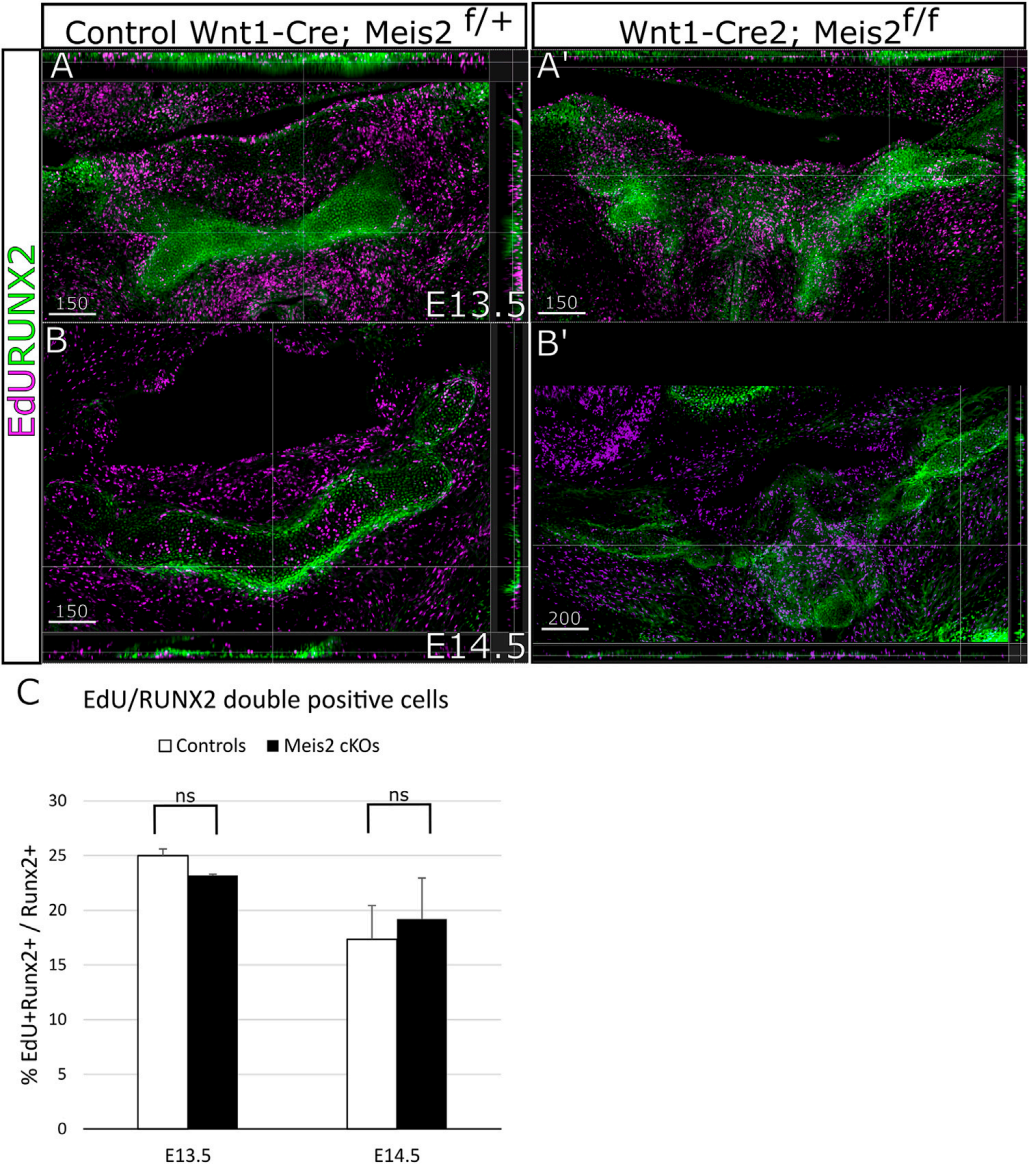
Unaltered proliferation of RUNX2 cells in Wnt1-Cre2; *Meis2* ^f/f^. **(A–B′)** Frontal frozen sections with orthogonal projections of hyoid regions in controls and Wnt1-Cre2; *Meis2* ^f/f.^ **(C)** Quantification of the percentage of double-positive EdU/RUNX2 cells in controls and Wnt1-Cre2; *Meis2* ^f/f^. The results were presented as mean ± SD. (E13.5 control 24.99 ± 0.62); (E13.5 Wnt1-Cre2; Meis2^f/f^ 23.16 ± 0.12); (E14.5 control 18.15 ± 4.05); (E14.5 Wnt1-Cre2; Meis2^f/f^ 20.87 ± 4.11). Statistical analysis was performed from biological triplicates for each genotype using unpaired two-tailed Student’s t-test. Scale bars in μm.

Concurrently, we focused on cell proliferation in the perichondrium by analysis of EdU incorporation in RUNX2^+^ perichondrium lining. Figure 6 shows that RUNX2 antibody reliably labels the perichondrium and that the perichondrium contains proliferating cells (approximately 25%) (Figure 6A-B′). Nonetheless, we found no statistical difference in perichondrial cell proliferation between controls and *Meis2* cKOs, neither at E13.5 nor at E14.5. Quantifications from orthogonal projections of three mutants and controls are summarized in Figure 6C. Our results indicate that abnormal growth in the mutant hyoid apparatus occurs at E13.5 and E14.5, and is caused by elevated proliferation of chondrocytes within the cartilage.

**FIGURE 7:**
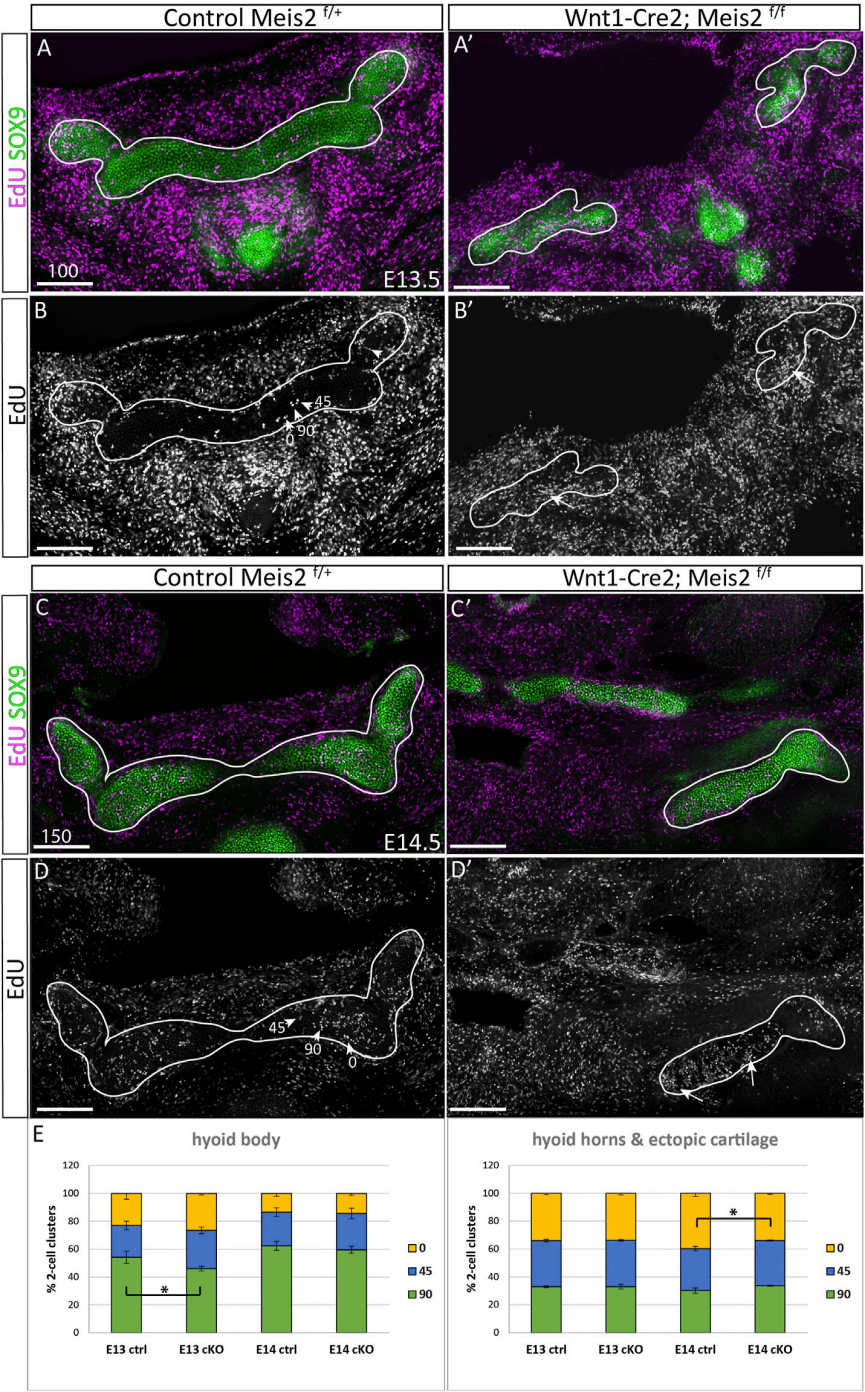
Irregular spatial distribution of proliferating cartilage progenitors in Wnt1-Cre2; *Meis2* ^f/f^ ectopic cartilage. **(A–B′)** Frontal frozen sections labelled with EdU (24-h pulse) and SOX9 antibody at stages E13.5, and E14.5 **(C–D′)**. 2-Cell EdU^+^ clusters were quantified according to their orientation towards the longitudinal axis in three angles 90, 45, 0° (arrowheads in **B** and **D**). Note a higher number and irregular distribution of EdU^+^ dividing cartilage progenitors in the aberrant cartilage (white arrows). **(E)** Quantifications of EdU^+^ doublets were made separately in the hyoid body and hyoid horns together with the ectopic cartilage in mutants. Statistical analysis was performed from biological triplicates for each genotype using unpaired two-tailed Student’s t-test. Scale bars in μm.

Kaucka et al., 2017 hypothesize that longitudinal growth and control of cartilage geometry are achieved by continuous generation of new condensations providing new clones that intercalate within existing columns. Such clones are oriented perpendicularly to longitudinal growth of the cartilage. We therefore concentrated on the position and orientation of clusters of proliferating cells. Spatial distribution of EdU^+/^SOX9^+^ double-positive cells was compared between controls and *Meis2* cKOs at E13.5 and E14.5 (Figure 7). We assessed orientation of 2-cell clusters in three arbitrary angles relative to the longitudinal axis (90, 45, 0°), and separately in the hyoid body and hyoid horns. The hyoid cartilage primordium contained clusters of dividing progenitors that consist of two cells, or four cells that were regarded as two 2-cell clusters for quantification. At E13.5, 54% (+/-4) of cell doublets in the hyoid body were oriented perpendicularly to longitudinal growth while remaining clusters were oriented in other angles (arrowheads in Figures 7B,D, quantification in E). Interestingly, the hyoid body in *Meis2* cKO at E13.5 significantly less perpendicular doublets (46% +/-2, *p*-value 0.0225). At E14.5, 62% (+/-3) EdU^+^ in controls and 60% (+/-3) in *Meis2* cKO displayed perpendicular orientation, showing no difference at this stage. On the other hand, hyoid horns in controls and in the ectopic cartilage of *Meis2* cKO did not display any tendency to perpendicular cluster orientation. Cell doublets in control horns were rather oriented in parallel with the longitudinal axis (40% +/-1), while remaining clusters were distributed stochastically (Figures 7A,D). The ectopic cartilage in *Meis2* cKO showed only stochastic orientation of EdU^+^ doublets (arrows in Figure 7B′,D′ and quantification in E). Altogether, zones of elevated proliferation were seen mostly at cartilage tips—in the horns and in the ectopic cartilage of *Meis2* cKO, in which the regular pattern of perpendicular orientation was not observed. This shows that the hyoid horns and the ectopic hyoid cartilage of *Meis2* cKOs consists of highly proliferating cells in uneven distribution. We suggest that the oriented growth is linked to the proliferation rate which may explain misshapen and ectopic cartilage in mutants.

### HAND2 and PBX1 expression domains are diminished in the hyoid region of *Meis2* cKOs

**FIGURE 8:**
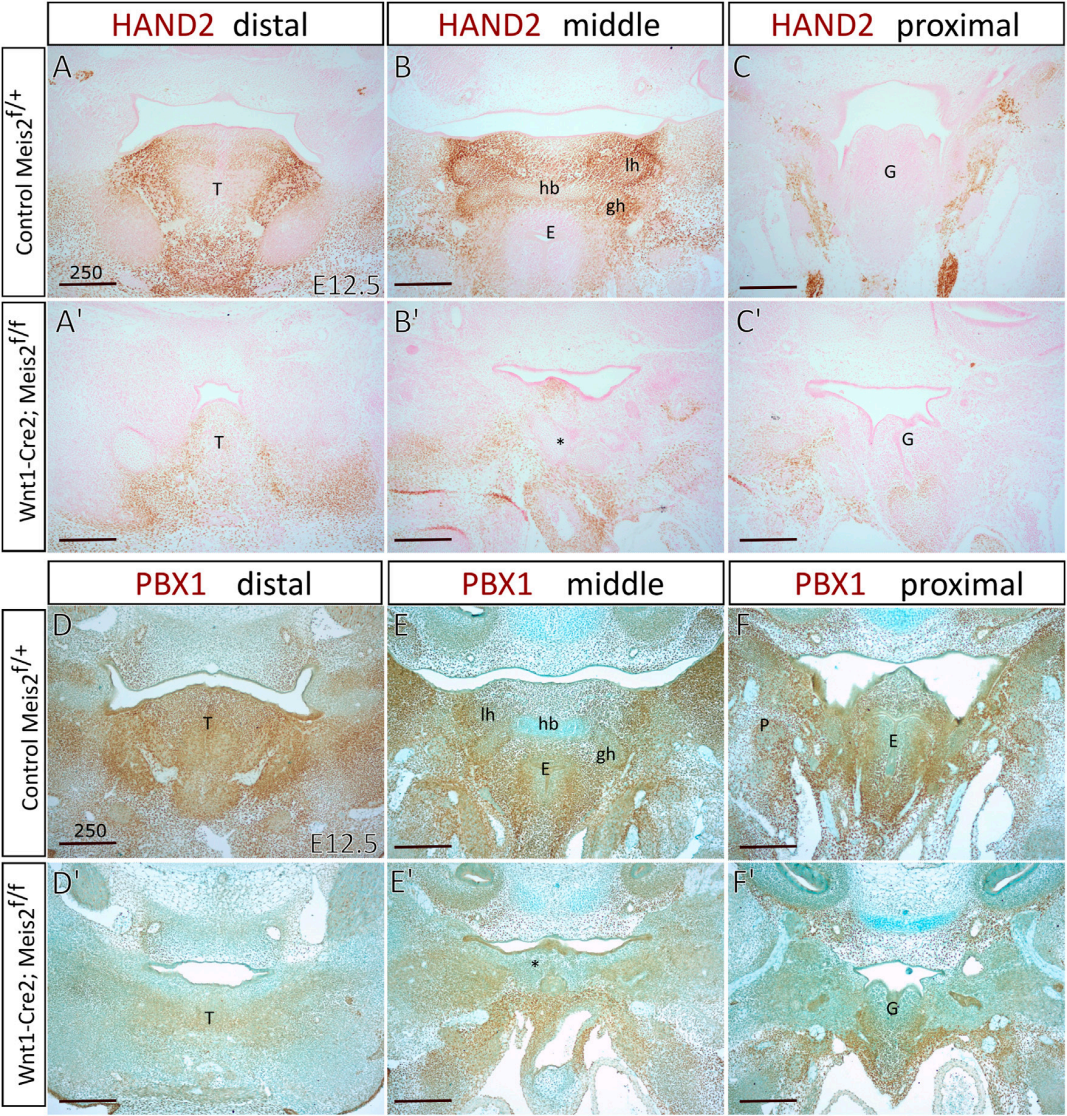
Diminished expression of *Hand2* and *Pbx1* in hyoid regions of Wnt1-Cre2; *Meis2* ^f/f^. Frontal paraffin sections of hyoid regions in E12.5 controls and Wnt1-Cre2; *Meis2* ^f/f^. **(A–C′)** The expression domain of HAND2 was diminished in E12.5 Wnt1-Cre2; *Meis2* ^f/f^ in the hyoid region, and the tongue. Sections were counterstained with nuclear fast red. **(D–F′)** The expression domain of PBX1 was diminished in the tongue, hyoid, and laryngeal regions in E12.5 Wnt1-Cre2; *Meis2* ^f/f^. Sections were counterstained with Alcian blue. Abbreviations: hb, hyoid body; gh, greater horns; lh, lesser horns; E, epiglottis; G, glottis; T, tongue; *—malformed hyoid primordium. Scale bars in μm.

Increased skeletogenesis in the hyoid apparatus probably originates from altered molecular pattern of the hyoid region. We monitored the expression of *Hand2* in the hyoid region in E12.5 embryos. Figures 8A′–C′ show that HAND2 protein almost disappeared in the tongue, hyoid and larynx region of *Meis2* cKO (4/4). This corresponds to reduced *Hand2* transcription in the distal-medial region of the mandibular process (Fabik et al., 2020). PBX1 often regulates transcription in the complex with MEIS factors. Moreover, *Pbx1* expression is down-regulated in palatal shelves of *Meis2* cKOs (Wang et al., 2020). Accordingly, we observed markedly reduced PBX1 signal in the hyoid region of E12.5 *Meis2* cKO embryos (4/4) (Figure 8D′–F′). We also noticed that the expression of PBX1 overlaps with the expression of MEIS2 in controls (compare Figure 1 and Figure 8).

**FIGURE 9:**
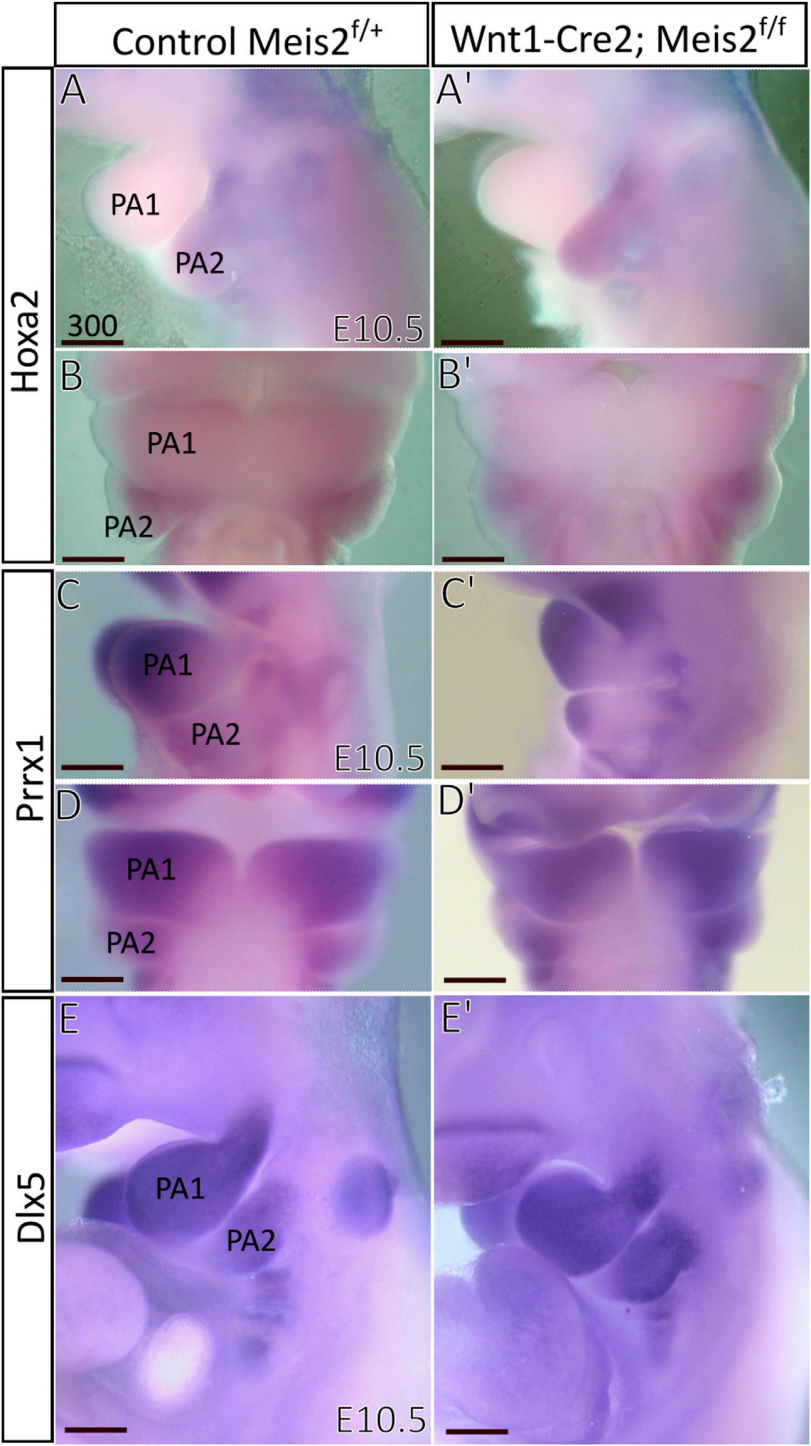
Unaltered expression of molecular markers in hyoid arches of Wnt1-Cre; *Meis2* ^f/f^. **(A–B′)** Expression domain of *Hoxa2* in E10.5 hyoid arch of control and *Meis2* cKO, lateral **(A,A′)** and frontal **(B,B′)** views. **(C–D′)** Expression domain of *Prrx1* in E10.5 pharyngeal arches in control and *Meis2* cKO, lateral **(C,C′)** and frontal **(D′-D)** views. **(E,E′)** Expression domain of *Dlx5* in E10.5 pharyngeal arches in control and *Meis2* cKO, lateral views. Scale bars in μm. Abbreviations: PA1, pharyngeal arch 1 (mandibular), PA2, pharyngeal arch 2 (hyoid).

Next, we focused on *Hoxa2*, which is widely considered a key determinant of the hyoid arch development. *Hoxa2* has been reported to be directly regulated by MEIS-PBX complex in zebrafish and lamprey (Parker et al., 2019, 2021) and MEIS2 protein is strongly expressed in the E10.5 hyoid arch (Fabik et al., 2020). However, we found no perceivable change in the expression of *Hoxa2* in E10.5 Meis2 cKOs (3/3) (Figure 9A–B′). *Prrx1* has been reported to have a similar pattern of expression to *Hand2* and *Prrx1*/*Prrx2* double nulls exhibit strikingly similar phenotype to *Meis2, Hand2 and Pbx1* mutants. We did not find, however, changes in *Prrx1* expression in E10.5 *Meis2* cKOs (3/3) (Figures 9C–D′). *Dlx5* is normally expressed in the mandibular (PA1) and hyoid (PA2) arches. Again, *Dlx5* expression was not altered in *Meis2* cKO (2/2) (Figure 9E,E′). Thus, we suggest that the initial molecular specification of the hyoid arch is established in E10.5 *Meis2* mutants. While MEIS2, PBX1, and HAND2 act together during hyoid development, *Hoxa2* expression is not controlled by MEIS2 alone.

## Discussion

Our work shows that the transcription factor MEIS2 regulates development of the hyoid region. Interestingly, the expression of *Hoxa2,* a master regulator of the hyoid arch development (Kanzler et al., 1998)*,* remained unchanged in *Meis2* mutants. It has been proposed that *Hoxa2* controls development of the hyoid arch by restricting the chondrogenic domain and inhibiting bone formation (Kanzler et al., 1998). Therefore, we find no alteration of *Hoxa2* in *Meis2* cKOs intriguing, as lack of *Meis2* results in ectopic loci of mesenchymal condensation and disinhibition of skeletogenesis.

Parker et al., 2019 identified consensus binding sites for MEIS and PBX in the NC-specific *Hoxa2* enhancer in lamprey, zebrafish, and mouse, suggesting direct regulation of *Hoxa2* expression by MEIS factor. Also, MEIS2 has been shown to cooperate with HOXA2 to instruct the identity of the murine hyoid arch (Amin et al., 2015). In fact, HOXA2 enhances MEIS binding to fine-tune molecular patterning of the hyoid arch. However, our results show that lack of *Meis2* alone does not alter the basic molecular identity of the hyoid arch.

MEIS and PBX form complexes that are critical for control of HOX-regulated genes (Choe et al., 2009; Longobardi et al., 2014). Our experiments revealed that PBX1 was reduced in the hyoid region of *Meis2* cKO. This was an expected result, as Selleri et al., 2001 observed the aberrant hyoid apparatus in *Pbx1* mutants that was similar to the one in *Meis2* cKO. Similarly, to *Meis2* mutants, there was no major change in the expression of *Hoxa2* in *Pbx1* knockouts (Selleri et al., 2001). Since the expression of *Hoxa2* is unaltered in both *Meis2* and *Pbx1* mutants, we ponder whether MEIS2 can substitute for PBX1 in *Hoxa2* regulation, and vice versa. It is also possible that the residual expression of *Pbx1* in *Meis2* cKOs and the presence of other PBX factors is sufficient to sustain the normal expression of *Hoxa2* and thus maintain the basic molecular identity of the hyoid arch in E10.5 *Meis2* cKOs. That would mean that the hyoid phenotypes in both *Meis2* and *Pbx1* knockouts are not a result of *Hoxa2* gene dose alteration. Since *Meis1* is also weakly expressed in the hyoid arch, we cannot exclude the possibility that *Meis2* is complemented by *Meis1* during primary molecular specification of the hyoid arch. Another reason for why the expression of *Hoxa2* was unaltered in *Meis2* cKOs could be that in this particular case, MEIS and PBX exert their functions independently from HOXA2.

*Pbx1* has been shown to be involved in chondrocyte maturation (Selleri et al., 2001). The histological analysis of the hyoid region in E16 *Pbx1* mutants showed an elongated aberrant cartilage consisting of small proliferating tightly packed chondrocytes, in place of the lesser horns of the hyoid. Selleri et al., 2001 further suggested that the aberrant cartilage in *Pbx1* mutants could have been the result of enhanced chondrocyte proliferation. Our data from *Meis2* cKOs are similar, further supporting the idea that MEIS2 acts upstream of PBX1 and MEIS/PBX complex controls chondrocyte proliferation in the hyoid region. Interestingly, *Pbx1* appears to serve an opposite function in limbs and ribs, as lack of *Pbx1* in ribs showed reduction in proliferation of chondrocytes, and precocious bone formation (Selleri et al., 2001). Research *in vitro* shows that PBX1 represses osteoblastogenesis by blocking HOXA10-mediated recruitment of chromatin remodeling factors and depletion of PBX1 increases osteoblast-related genes, histone acetylation and CBP/p300 recruitment (Gordon et al., 2010). Unlike *Meis2* cKOs, *Pbx1* mutants show no signs of endochondral ossification in the aberrant skeletal chain, although skeletal preparations of E17.5 *Pbx1* mutants would be needed to conclusively confirm this notion. Interestingly enough, *Meis2* cKOs have also decreased *Pbx1* expression in the secondary palate, as MEIS2 binds to specific loci in *Runx2*, *Pbx1*, *Shox2,* and other osteogenic genes, to regulate their expression in the secondary palate (Wang et al., 2020).

Despite the fact that Selleri et al., 2001 identified the hyoid phenotype in *Pbx1* mutants as a homeotic transformation akin to *Hoxa2* mutant phenotype, we suggest that *Pbx1* mutant phenotype is actually more similar to *Meis2* mutant phenotype. We presume that *Meis2* and *Pbx1* mutants do not exhibit homeotic transformation and that the aberrant hyoid apparatus is a consequence of ectopic mesenchymal loci and enhanced chondrocyte proliferation. Therefore, we propose that hyoid phenotypes in *Meis2* and *Pbx1* mutant share a similar mechanistic origin.

*Hand2* is thought to control development of ventral regions of the PAs, and ventral region of the hyoid arch is represented by the lesser horns of the hyoid bone. We found *Hand2 e*xpression to be reduced in the hyoid region of *Meis2* cKOs. Previously, we reported that reduced expression of *Hand2* in the medial region of the mandibular process in *Meis2* mutants led to bone formation at the expense of tongue organogenesis (Fabik et al., 2020). Thus, morphological defects in mandibles and tongues of *Meis2* cKOs are most probably the result of impaired molecular patterning of the mandibular arch as early as at E10.5. We suggest that *Hand2* plays a similar role in the hyoid arch, i.e., *Hand2* specifies molecular domains of the hyoid arch and controls subsequent cell fate decision in differentiating neural crest cells. Therefore, as *Hand2* suppression of osteogenesis in ventral mandibular arch allows formation of the tongue connective tissue, so could *Hand2* suppress the formation of redundant cartilage in the hyoid region.

Unlike in *Meis2* mutants, no major change in the expression of *Hand2* was seen in *Pbx1* knockouts (Selleri et al., 2001). In contrast, mouse mutants overexpressing *Hand2* exhibit elevated levels of *Pbx1* and *Hand2* in the medial mandibular process and hyoid arch, and altered expression of these genes in the maxillary process (Funato et al., 2016). Furthermore, Osterwalder et al., 2014 reported that HAND2 chromatin complexes were enriched in the genomic region of *Pbx1*. Thus, HAND2 may directly regulate *Pbx1* during craniofacial development. In the zebrafish heart, Hand and Pbx1 have been implicated to act together (Maves et al., 2009), while in the mouse limb they appear to function in parallel pathways (Capellini et al., 2006). Consequently, the *Hand2* expression is not dependent on PBX in the mouse limb. All taken together, data from pharyngeal arch, palate, heart, and limb suggest that *Pbx1* acts downstream of both *Meis2* and *Hand2* in the hyoid region.

It should be noted that *Hand2* mutants develop various abnormal fusions in the hyoid apparatus. Conditional inactivation of *Hand2* in *Col2-Cre; Hand2* embryos revealed skeletal malformations of the primordia of the hyoid, thyroid, and cricoid cartilages, abnormal cartilaginous fragments in the middle ear region and extremely abnormal shape of middle ear bones, as well as most of the cranial base cartilage missing (Abe et al., 2010). Neural-crest specific deletion of *Hand2* leads to a similar set of anomalies, i.e., the aberrantly ossified hyoid body and lesser horns, the lesser horns fused to duplicated palatine bones, lesser horns forming articulations with the malformed malleal cartilage anlagen, and the styloid process articulating with the greater horns (Barron et al., 2011). Worth of note, the fusion of lesser horns and the styloid process, as the one seen in *Meis2* and *Pbx1* knockouts, has never been reported in neither *Hand* mutants.

*Hand2* mutant phenotype strongly supports the notion that *Hand2* acts to suppress formation of the cartilage in the hyoid region. Abe et al., 2010 reported that *Hand2* regulates endochondral ossification during limb skeletogenesis and also determines the site of chondrogenesis by outlining the region of the future cartilage template (Abe et al., 2010). In addition to chondrogenic regulation, HAND proteins bind to RUNX2 and are responsible for repression of RUNX2 activity (Funato et al., 2016). All in all, *Hand2* shows a major role in chondrogenic and osteogenic repression. Therefore, we suggest that *Meis2* mutant phenotype could at least partially be caused by decreased levels of *Hand2* during osteochondrogenesis.

*Prrx1*/*Prrx2* double mutants exhibit a surprisingly similar phenotype to *Meis2*, *Pbx1*, and *Hand2* mutants (ten Berge et al., 2001). *Prrx1/Prrx2*, *Meis2*, and *Pbx1* mutants grow the aberrant skeletal chain that stretches from the styloid process to the lesser horns of the hyoid (ten Berge et al., 2001; Selleri et al., 2001). Additionally, an overabundance of RUNX2^+^ cells in *Prrx1/Prrx2* mutants indicates precocious or accelerated osteogenesis (Balic et al., 2009). Even the domains of *Prrx1* expression in ventral regions of the mandibular and hyoid arches are similar to the corresponding expression domains of *Hand2*. In spite of that, we found no alteration in the expression of *Prrx1* in *Meis2* mutants. Similarly, no changes in the expression of *Prrx1* were reported in *Hand2* mutants lacking the ventrolateral branchial enhancer (Barbosa et al., 2007). Thus, neither *Meis2* elimination nor *Hand2* down-regulation do not seem to alter the overall expression of *Prrx1* in the E10.5 hyoid arch. Correspondingly, *Prrx1*/*Prrx2* double null mutants do not show any changes in *Hand2* expression (Balic et al., 2009). Given conspicuous similarities between *Prrx1*/*Prrx2*, *Meis2*, *Pbx1,* and *Hand2* mutants, one would expect their interaction during the hyoid development. Nonetheless, PRRX1 may not be a critical part of the MEIS2-HAND2-PBX1 circuit and may act independently in the hyoid development.

We presume that mineralization of the caudal part of the aberrant skeletal chain is caused by misspecification of cartilage, followed by commitment of cartilage cells to the osteoblast lineage. The mineralization is confined solely to the small caudal part of the chain and does not extend cranially to the styloid process or caudally to the hyoid horns. Mineralization of the small part of the aberrant skeletal chain in *Meis2* cKOs takes place at the stage in which only the hyoid body is supposed to be ossified. Therefore, we assume that the caudal part of the aberrant skeletal chain is incorrectly specified, resulting in aberrant bone formation via endochondral ossification. According to our observations, no other parts of the hyoid apparatus in *Meis2* cKOs show signs of cartilage misspecification.

We find that there are several mechanisms of growth in the hyoid cartilages that could be possible. There are no plate-like zones in pharyngeal cartilages (Kaucka et al., 2017), but the hyoid horns are normally united with the body either by fibrous tissue or synovial joints. We consider it possible that these joints contain stem cells that contribute to growth of the hyoid structure. Alternatively, the hyoid horns could grow in a manner similar to rod-shaped cartilage (i. e., Meckel’s cartilage), since they originate from pharyngeal cartilages. Unlike the horns, the body originates from *de novo* mesenchymal condensation in the midline. Therefore, the hyoid body could possess a different growth mechanism to the hyoid horns.

The role of perichondrial cells during cartilage expansion is still unclear, although some studies showed that the perichondrium represented a source of chondrocytes and osteoblasts **(** Maes et al., 2010; Kobayashi et al., 2011; Li et al., 2017). In addition, Kaucka et al., 2017 showed a clonal relationship between columns of chondrocytes and perichondrial cells. In *Meis2* cKOs, we observed no difference in RUNX2^+^ proliferating cells in comparison with controls. However, we found statistically significant difference in SOX9^+^ proliferating cells in *Meis2* cKOs when compared to controls. This finding shows that abnormally elevated proliferation in *Meis2* cKOs takes place within the hyoid cartilages but not in the hyoid perichondrium. Our data suggest that all parts of the hyoid apparatus form simultaneously. Therefore, we do not think that hyoid horns are formed via intercalation of mesenchymal condensations into the existing hyoid body cartilage, or vice versa. The aberrant skeletal chain between the styloid process and the lesser horns also appears to form at the same time and not sequentially. Therefore, we do not think that the aberrant cartilage is actually an elongated tip of either the lesser horn or the styloid process. Kaucka et al., 2017 observed the vast majority of dividing cells in perpendicular or transverse orientation in sheet-shaped or rod-shaped facial cartilage, respectively. We found a similar pattern in the hyoid cartilage only in 54% of EdU^+^ doublets at E13.5 and 62% at E14.5. We presume that fast growing zones display less regular orientation of proliferating chondroprogenitors.

**FIGURE 10:**
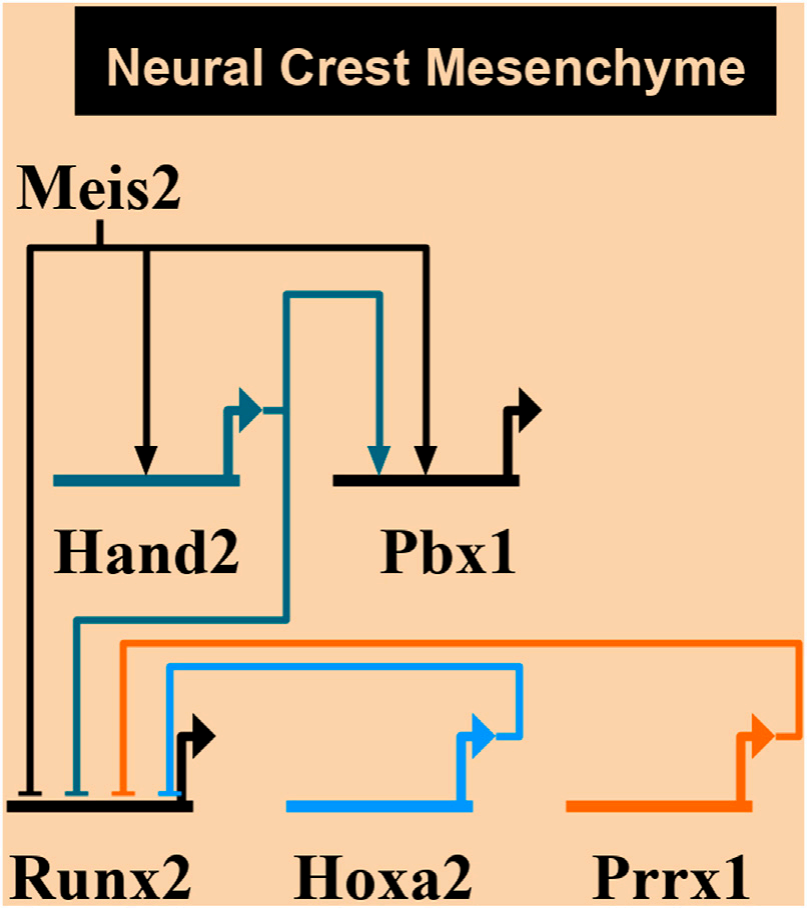
A proposed network of transcription factors converging to MEIS2. MEIS2**-**HAND2-PBX circuit controls skeletogenesis in mandibular and hyoid arches by restricting skeletogenic domains. On the other hand**,** MEIS2, HOXA2, and PRRX1 are not directly linked during the hyoid development. Created using Biotapestry (Longabaugh et al., 2005, 2009; Paquette et al., 2016).

Figure 10 summarizes a proposed network of transcription factors converging to MEIS2. In the mandibular arch, MEIS2 controls the expression of *Hand2* and *Pbx1*, as shown by diminished domains of both HAND2 and PBX1 in lingual mesenchyme in Figure 8 (see also Fabik et al., 2020). MEIS2 and HAND2 control *Pbx1*, as suggested by studies concerning development of pharyngeal arches, palate, heart, and limbs (Capellini et al., 2006; Maves et al., 2009; Funato et al., 2016; Wang et al., 2020). At the same time, MEIS2 affects *Runx2*, as lack of MEIS2 in NCCs results in expansion of the RUNX2^+^ domain in the mandibular arch (Fabik et al., 2020) and in palatal shelves (Wang et al., 2020). HAND2 also controls the expression of Runx2, as shown by studies concerning development of the medial-distal tip of the mandibular arch (Funato et al., 2009; Fabik et al., 2020). Similarly, Prrx1 affects *Runx2*, as shown by expansion of the RUNX2^+^ domains in *Prrx1* mutants (Balic et al., 2009). We propose here that MEIS2 controls the expression of *Hand2* and *Pbx1* also in the hyoid arch. HOXA2, as a key determinant of molecular patterning of the hyoid arch, has also been implicated to inhibit bone formation (and *Runx2*) during normal hyoid development (Kanzler et al., 1998). However, we found no change in the expression of neither *Hoxa2* nor *Prrx1* in *Meis2* cKOs, so we presume that MEIS2, HOXA2 and PRRX1 are not directly linked during the hyoid apparatus development. Therefore, we propose an additional regulatory network MEIS2-HAND2-PBX1 that controls skeletogenesis by restricting Sox9^+^ and Runx2^+^ domains in the mandibular and hyoid arches.

In *Meis2* cKOs, we observed mesenchymal condensations forming ectopically in multiple places simultaneously. Based on our data, we are convinced that multiple mesenchymal condensations form between the styloid process and the lesser horns. They differentiate and proliferate, fuse and finally give rise to the aberrant skeletal chain. Alternatively, elevated proliferation of chondrogenic precursors in *Meis2* cKOs could ensure that ectopic mesenchymal loci emerging earlier reach a critical size required for chondrogenic differentiation. Afterwards, hyoid cartilages seem to grow normally via clonal columns. The abnormal shape of the hyoid bone, including a midline body projection, could also be the result of elevated proliferation. All in all, we presume that lack of *Meis2* results in ectopic placement of mesenchymal condensations and elevated proliferation within the cartilage and chondrogenic condensations, which together result in formation of the aberrant hyoid apparatus. This defect originates in early chondrogenic stages of development of facial skeleton. Similar to Rodríguez-Vázquez et al., 2006; 2015, we found no evidence that cartilage develops in the central part of the hyoid arch under normal conditions. The embryological theories consider two scenarios in which Reichert’s cartilage forms the stylohyoid ligament: 1) Reichert’s cartilage degenerates and leaves behind its fibrous sheath producing the stylohyoid ligament; or 2) perichondrium of Reichert’s cartilage serves as a guide for formation of the stylohyoid ligament. Both seem incorrect in light of recent data (Rodríguez-Vázquez et al., 2006; Rodríguez-Vázquez et al., 2015)—there is no central Reichert’s cartilage that could give rise to the stylohyoid ligament. Therefore, as for the etiology of congenital variants of the aberrant hyoid apparatus and the Eagle’s syndrome, we assume that the possibility of persistence of Reichert’s cartilage or its precursors in the stylohyoid ligament is minimal. We think that the congenital variants of the aberrant hyoid apparatus and the Eagle’s syndrome develop as *de novo* chondrogenic condensations. Alterations in MEIS2-HAND2-PBX1 regulatory circuit during hyoid development could stand behind congenital hyoid pathologies in humans. This indicates shared molecular mechanisms controlling skeletogenesis of the mandibular process and the hyoid arch, which should be considered in a clinical practice.

## Data Availability

The original contributions presented in the study are included in the article/Supplementary Material, further inquiries can be directed to the corresponding author.
